# Immune-Challenged Fish Up-Regulate Their Metabolic Scope to Support Locomotion

**DOI:** 10.1371/journal.pone.0166028

**Published:** 2016-11-16

**Authors:** Camille Bonneaud, Robbie S. Wilson, Frank Seebacher

**Affiliations:** 1 Centre for Ecology & Conservation, University of Exeter Penryn Campus, Penryn TR10 9FE, Cornwall, United Kingdom; 2 Station d’Ecologie Expérimentale du CNRS, USR 2936, 09200 Moulis, France; 3 School of Biological Sciences, University of Queensland, Brisbane St Lucia QLD 4072, Australia; 4 School of Biological Sciences, University of Sydney, Sydney NSW 2006, Australia; Universidad de Granada, SPAIN

## Abstract

Energy-based trade-offs occur when investment in one fitness-related trait diverts energy away from other traits. The extent to which such trade-offs are shaped by limits on the rate of conversion of energy ingested in food (e.g. carbohydrates) into chemical energy (ATP) by oxidative metabolism rather than by the amount of food ingested in the first place is, however, unclear. Here we tested whether the ATP required for mounting an immune response will lead to a trade-off with ATP available for physical activity in mosquitofish (*Gambusia holbrooki*). To this end, we challenged fish either with lipopolysaccharide (LPS) from *E*. *coli* or with Sheep Red Blood Cells (SRBC), and measured oxygen consumption at rest and during swimming at maximum speed 24h, 48h and 7 days post-challenge in order to estimate metabolic rates. Relative to saline-injected controls, only LPS-injected fish showed a significantly greater resting metabolic rate two days post-challenge and significantly higher maximal metabolic rates two and seven days post-challenge. This resulted in a significantly greater metabolic scope two days post-challenge, with LPS-fish transiently overcompensating by increasing maximal ATP production more than would be required for swimming in the absence of an immune challenge. LPS-challenged fish therefore increased their production of ATP to compensate physiologically for the energetic requirements of immune functioning. This response would avoid ATP shortages and allow fish to engage in an aerobically-challenging activity (swimming) even when simultaneously mounting an immune response. Nevertheless, relative to controls, both LPS- and SRBC-fish displayed reduced body mass gain one week post-injection, and LPS-fish actually lost mass. The concomitant increase in metabolic scope and reduced body mass gain of LPS-challenged fish indicates that immune-associated trade-offs are not likely to be shaped by limited oxidative metabolic capacities, but may instead result from limitations in the acquisition, assimilation or efficient use of resources.

## Introduction

Pathogens can represent intense selection pressures for their hosts [1, 2]. Despite this, the evolution of host resistance through the activation of a protective immune response may be constrained by the energetic costs of immune functioning and trade-offs with other fitness-related traits [3–6]. A growing number of studies have provided evidence of costly immune responses to inert antigens, with immune-challenged individuals showing increased nutritional intake [7, 8] and body mass loss [9, 10] compared to controls. Because hosts usually have access to limited resources, the energy allocated to fuel immune responses may be diverted away from other fitness-related functions, such as growth, reproduction, and maintenance [3]. Accordingly, immune challenges have been shown to be associated with reduced locomotor performance [11–13] and lowered investments in reproduction and growth [14, 15]. However, while energetic constraints are often assumed to mediate life history trade-offs [3], the physiological processes by which this might occur still remain to be elucidated.

Previous studies quantifying the energetic cost of immunity have focused on measuring changes in basal metabolic rates following immune challenge [9, 16, 17]. Basal (or resting when measured under less restrictive circumstances) metabolic rates reflect the rate of ATP hydrolysis required to maintain cellular processes during physical inactivity [18]. For instance, vaccinated rainbow trout (*Oncorhynchus mykiss*) displayed higher routine metabolic rates (measured in unfed fish spontaneously swimming and which can be indicative of basal metabolism) than controls [19], with rates increasing by ~20% 223° days post-vaccination (equivalent to 22.3 days at 10°C) [20]. Similarly, four species of insects (*Tenebrio molitor*, *Acheta domesticus*, *Cotinis nitida* and *Periplaneta americana*) showed an increase in metabolic rate by up to 28% following the induction of an innate immune encapsulation response, during which hemocytes form a multi-layered sheath around a parasite or antigen [21]. Cutrera et al [22] reported that tuco-tucos (*Ctenomys talarum*) experimentally-challenged with Sheep Red Blood Cells (SRBC) experienced a 20–35% increase in metabolic rate, which was equivalent to a 15% increase in daily energetic expenditure. Martin et al. [23] and Eraud et al. [24] found that the increase in basal metabolic rate displayed by collared doves (*Streptopelia decaocto*) and house sparrows (*Passer domesticus*) following injections with inert antigens was of a magnitude similar to that required to produce half an egg per day and to maintain an optimal body temperature at an ambient temperature 1–2°C below thermo-neutrality, respectively. While immune responses have thus been shown to increase the resting metabolic rate, it remains to be determined whether such immune-triggered increase in ATP use at rest means that less ATP is available for engaging in other aerobically-challenging activities.

The level of energy that an individual can allocate to fitness-related activities such as reproduction, foraging and engaging in behavioural interactions, can be estimated by the aerobic metabolic scope [25–27]. The aerobic metabolic scope is defined as the difference between the maximal metabolic rate, which reflects maximal mitochondrial flux [25], and the resting metabolic rate, and therefore represents the metabolic capacity to engage in aerobically-challenging activities [28]. Metabolic scopes may decline either when there is an increase in the resting metabolic rate or when there is a decrease in the maximal metabolic rate. Hence, if metabolic maintenance costs increase, the resultant reduction in metabolic scope can create an allocation trade-off [29]. Decreases in the amount of energy available to other activities may, however, be counterbalanced physiologically through an increase in the capacities of mitochondrial metabolic pathways [27, 30, 31]. While such compensatory responses may abolish ATP shortages, trade-offs may still persist if investment in multiple aerobically-challenging activities depletes storages of ingested chemical energy with detrimental consequences for body condition.

Here we examined the energetic basis of immune-associated resource allocation trade-offs in wild-caught, female mosquitofish (*Gambusia holbrooki*). Individuals were challenged either with lipopolysaccharides (LPS) isolated from *Escherichia coli* or with Sheep Red Blood Cells (SRBC) to investigate how differences in the type of immune response elicited may constrain energetic investments in other functions [22, 32–34]. Although fish are thought to be less sensitive to endotoxins such as LPS than other vertebrates [35–37], LPS-challenges in fish *in vivo* and *in vitro* nevertheless induced a strong inflammatory response [38, 39], and increased the production of cytokines and acute phase proteins, as well as stimulated T and B lymphocytes, macrophages and complement systems [26]. For example, the expression of cytokines in a monocyte-macrophage lineage of rainbow trout was detectable 6h after exposure to *E*. *coli* LPS and increased over 24h [30, 33]. LPS also induces antibody production, which in the brown trout (*Salmo trutta*) was detectable on day 14 post-injection [40]. However, antigen-binding and antibody-secreting cells were detected in the spleen and kidney of these fish as early as 2 and 4 days after injection, with peaks reached between day 14 and 18 post-injection [41]. SRBC, on the other hand, induced a non-pathogenic T and B-cell dependent antibody response [32]. Brown trout immunized with SRBC displayed detectable levels of antigen-binding and antibody-secreting cells on day 6 post-injection, with levels peaking on day 12 [41]. Levels of antibody-producing cells were detectable as early as day 5 and peaked on day 10 in the Mozambique tilapia (*Oreochromis mossambicus*) [42].

Based on these studies, we can make the following predictions. First, we predicted that if immune responses were energetically costly, then immune-challenged individuals would exhibit higher resting metabolic rates than controls. Second, we tested whether limits on ATP production and the amount of ATP available can give rise to immune-associated trade-offs. We predicted that, if this were the case, immune-challenged individuals should experience a reduction in metabolic scope relative to controls. Conversely, if immune-associated trade-offs are shaped by a limited availability of ingested energetic resources, then immune activation should instead be associated with a reduction in body mass.

## Material and Methods

### Study animals

All procedures were approved by a prefectorial order from Ariège, France (Agréments de l'établissement pour l’élevage et l'expérimentation no. 0108 and no. SA-013-PB-092; certificats d'autorisation d’élevage et d'expérimentation sur poissons vivants to Oliver Guillaume, no. 09–273 and no. A09-3) and by the University of Sydney Animal Ethics Committee (approval no. L04/10-2010/3/5411). Wild mosquitofish were captured in July 2011 from natural ponds on private land near Perpignan (42.698°N, 2.895°E) in southern France, with permission from local landowners. This invasive species needs no permit for collection. Animals were immediately brought back to the laboratory at the Station d’Ecologie Expérimentale du Centre National de la Recherche Scientifique (CNRS) in Moulis (Ariège, France), where they were housed in large containers (100 fish at approximately 1 fish/L), with a mixture of water from their capture sites and aged tap water. The females used in this study were housed individually (in 300×250×200 mm tanks) so that we could follow individuals. Females were habituated to their individual tanks for two weeks before the start of the experiment. At the time of capture, water temperature at the sites of capture varied between 28–30°C; fish were therefore kept at 30°C in the laboratory throughout the habituation and experimentation phases. Because female mosquitofish store sperm and will be mated immediately upon reaching maturity [20], we categorized females based on the shape and distension of their abdomen to ensure that all individuals used were at a similar early stage of pregnancy (for detailed methods see [43]) [27]. Fish were fed to satiety with commercial fish flakes once per day. Food was withheld 24 h prior to immune-treatment and prior to all measurements of metabolic rates. This ensured that fish were in a post-absorptive state and therefore fit the requirement for basal metabolic rate measurement (i.e., calm, motionless and post-absorptive). Throughout the experiment, fish were monitored daily for signs of stress (e.g., gasping at the surface, loss of appetite, unusual swimming patterns and symptoms of disease), in which case they would have been immediately euthanized by slowly mixing an appropriate dose of clove oil in water to the fish tank. None of the fish was euthanized during the course of the experiment, however, and all fish were euthanized with a lethal dose of clove oil at its conclusion.

### Immune challenge

After approximately two weeks in captivity, females were randomly assigned to experimental or control groups (experimental LPS-injected: N = 10; experimental SRBC-injected: N = 9; controls: N = 10). Individuals were challenged with an intra-peritoneal injection of LPS (Sigma; St. Louis, USA; L4005; 10μg/g fish at 1g/L [44]) or SRBC (Sigma R3378; 20μg/g fish at 1g/L [42, 45]) for experimental treatments, and 0.01mL of saline solution (PBS) for controls. Prior to injection, all fish were lightly anesthetized in a clove oil solution, and after injection animals were placed immediately in aged tap-water where they recovered within 1–2 minutes. At the onset of the experiment we measured both total body length ((±0.05 mm) and body mass (hereafter: initial mass, ±0.01 g), although body mass was again obtained at the end of the experiment after 7 days (~168 hours). Body length and body mass were highly correlated at experiment onset (Correlation coefficient, r_p_ = 0.97, p < 0.001).

### Oxygen consumption

In fish, the immune response to LPS is known to occur within hours of injection and last for at least 2 weeks [30, 33, 40]. On the other hand, antibody responses to SRBC can become detectable 5–6 days post-immune challenge and peak 10–12 day post-injection [41, 42]. Consequently, we measured oxygen consumption to estimate resting and maximal metabolic rates at 24h, 48h and 168h (7 days) post-challenge in LPS-exposed individuals and control fish, and at 7 days only in SRBC-exposed ones. Oxygen consumption is a commonly-used, indirect measure of metabolic rates [46]. Oxygen consumption was measured according to published methods [43] with a fibre-optic oxygen system (Fibox 3, Presens, Regensburg, Germany) monitoring sensor spots (Presens, Germany) attached to the insides of respirometers according to the manufacturers’ instructions.

We first measured resting oxygen consumption by allowing fish to rest in a cylindrical glass respirometer (245 ml volume) placed into a darkened tank for 45–60 minutes. We then sealed the respirometer, making sure not to disturb the fish, and let the fish rest for a further 10–15 minutes before recording the decrease in oxygen over a 7–10 minute period or until a steady rate of oxygen decrease was established; we followed the decrease in oxygen levels within the respirometer in real-time.

Maximal rates of oxygen consumption were determined immediately after in a cylindrical glass respirometer (415 ml) placed on a magnetic stirrer [43]. A magnetic stirbar within the respirometer created water flow that could be adjusted with the control on the magnetic stirrer. Turbulence and eddies within the respirometer were minimised by a central column suspended from the lid. Fish were placed into the respirometer and the speed was increased slowly until fish swam steadily, but occasionally had to struggle to maintain their position in the water column, i.e. fish occasionally went backwards in the water column and had to engage in burst swimming to regain their position, indicating near maximal swimming speeds. Oxygen consumption was measured during 6–8 minutes of swimming at that speed. Rates of oxygen consumption (in μmol g^-1^ min^-1^) were determined as the slope of the decrease in oxygen content divided by the fish body mass and multiplied by the volume of the container [46]. We calculated exercise-induced metabolic scope as the difference between resting and swimming oxygen consumption.

### Statistical analyses

All statistical analyses were performed using SAS software version 9.3 (SAS Inc., Cary, NC). When required, data were log_10_-transformed before analyses to fulfil assumptions of normality and homoscedascity. Because metabolic rates are generally influenced by body mass (Hill et al 2012), we initially tested whether this was the case in our study using three regression analyses in which body mass was regressed against, respectively, resting and maximal metabolic rates, as well as metabolic scope. We restricted these analyses to those in control groups, calculated averages in metabolic parameters over the measures obtained at 24h, 48h and 168h (7 days) post-challenge. We then tested whether resting and maximal metabolic rates, and metabolic scopes of LPS-injected fish differed from those of controls using multivariate general linear mixed models with treatment (LPS or PBS), time, and their interaction, and with initial mass as fixed effects, and with individual as the random effect; for each time point, differences between LPS-challenged and control fish were contrasted within the same model using the “estimate” statement. Non-significant interactions were removed from final models. To examine how SRBC-injected fish differed in their resting and maximal metabolic rates and in their metabolic scope relative to controls and to LPS-fish, we conducted general linear models with treatment (SRBC, LPS or PBS) and initial mass as fixed effects; between-treatment differences were contrasted within the models using the “estimate” statement. Treatment effects on changes in body mass over the course of the experiment were conducted using a general linear model with the difference in body mass between 0 and 7 days post-injection as the dependent variable and with treatment and initial mass as fixed effects; between-treatment differences were contrasted using the “estimate” statement.

## Results

### Effects of immune treatment on metabolic rates

In control individuals, mass had significant (or nearly significant) negative effects on resting and maximal rates of oxygen consumption (resting: F_1,8_ = 8.69, p = 0.018; estimate(±SE) = -0.11±0.036; maximal: F_1,8_ = 4.97, p = 0.057; estimate(±SE) = -0.23±0.10); while mass had little effect on metabolic scope (F_1,8_ = 0.88, p = 0.38; estimate(±SE) = -0.12±0.13). We thus included body mass at experiment onset as a covariate in all analyses investigating the metabolic cost of immune activation.

**Fig 1:**
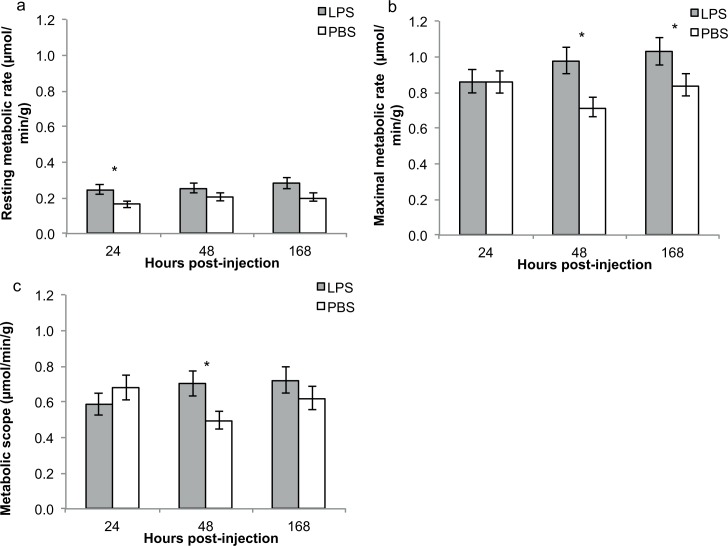
Rates of oxygen consumption and metabolic scope of LPS-injected and PBS (control) fish. a: Resting and b: maximal rates of oxygen consumption, and c: metabolic scope, 24h, 48h and 168h (7 days) after treatment. Values show predicted means (in μmol/min/g) with standard errors (* indicates p<0.05).

LPS-injected fish displayed higher resting and maximal rates of oxygen consumption than saline-injected controls over the course of the experiment (GLMM; resting rates of oxygen consumption: treatment: F_1,38_ = 8.8, p = 0.005, time: F_2,38_ = 1.2, p = 0.32; initial mass: F_1,38_ = 9.32, p = 0.004; maximal rates of oxygen consumption: treatment: F_1,38_ = 6.14, p = 0.018, time: F_2,38_ = 1.1, p = 0.33, initial mass: F_1,38_ = 13.7, p<0.001; Table 1; Fig 1A and 1B). Separate analyses of each time point revealed significant between-treatment differences in resting metabolic rates 24 h post-challenge and in maximal metabolic rate 48 h post-challenge, and a marginally significant between-treatment difference in resting metabolic rate 7 days post-challenge (resting metabolic rate: 24 h: t_36_ = 2.41, p = 0.021; 48 h: t_36_ = 1.22, p = 0.230; 7 days: t_36_ = 1.97, p = 0.057; maximal metabolic rate: 24h: t_36_ = 0.02, p = 0.99; 48h: t_36_ = 2.88, p = 0.007; 7 days: t_36_ = 1.90, p = 0.066).

**Table 1:** Metabolic measures for immune-challenged (with LPS or SBRC) and control (saline-injected) mosquitofish.

Time post-treatment	Metabolic measure	Treatment		
		Control	LPS	SRBC
24h	Resting	0.163 ± 0.053	0.293 ± 0.174	
	Maximum	0.831 ± 0.147	0.932 ± 0.255	
	Scope	0.668 ± 0.147	0.640 ± 0.224	
48h	Resting	0.202 ± 0.060	0.282 ± 0.083	
	Maximum	0.692 ± 0.124	1.038 ± 0.198	
	Scope	0.490 ± 0.110	0.756 ± 0.207	
7 days	Resting	0.205 ± 0.078	0.322 ± 0.150	0.209 ± 0.082
	Maximum	0.809 ± 0.133	1.196 ± 0.675	1.696 ± 0.786
	Scope	0.605 ± 0.127	0.874 ± 0.579	1.488 ± 0.750

We provide raw means (in μmol/min/g) and standard deviations for resting and maximal metabolic rates and for metabolic scopes at 24h, 48h and 7 days post-challenge with LPS, and at 7 days post-challenge with SRBC.

**Fig 2:**
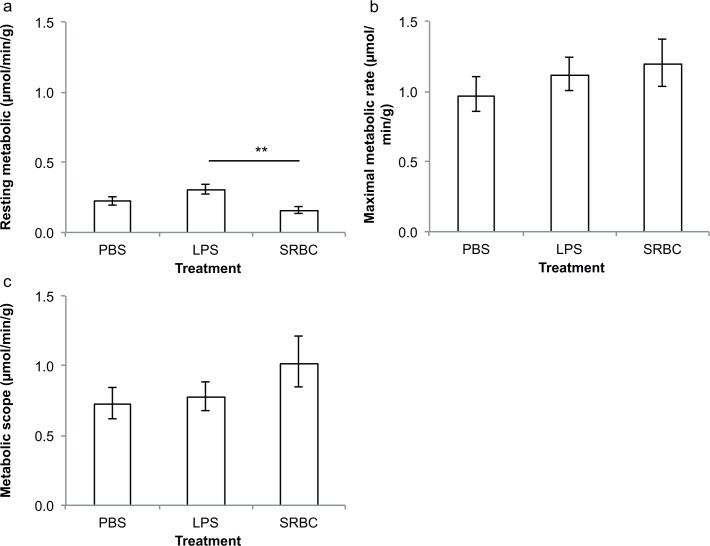
Rates of oxygen consumption and metabolic scope of SRBC-, LPS- and PBS-injected (control) fish. a: Resting and b: maximal rates of oxygen consumption, and c: metabolic scope, 7 days after treatment. Values show predicted means (in μmol/min/g) with standard errors (** indicates p<0.01).

We then examined the metabolic cost of an immune response to SRBC and compared the metabolic rates of SRBC- and LPS-fish. Resting rates of oxygen consumption 7 days post-injection differed significantly between treatments (GLM, treatment: F_2,25_ = 5.6, p<0.010, initial mass: F_1,25_ = 4.9, p = 0.036; Table 1; Fig 2A and 2B). However, between-treatment tests revealed that differences were significant between LPS and SRBC-fish only (t = 3.17, p = 0.004), with SRBC-fish displaying resting rates of oxygen consumption that were 50% lower than LPS-injected ones, but not between SRBC-fish and saline-injected controls (t = 1.4, p = 0.17). Treatment did not affect maximal rates of oxygen consumption 7 days post-injection (GLM, treatment: F_2,25_ = 0.5, p = 0.60, initial mass: F_1,25_ = 8.3, p = 0.008), with no significant difference detected between SRBC-fish and either LPS-fish or controls (p>0.1).

### Effects of immune treatment on metabolic scopes and body mass

**Fig 3:**
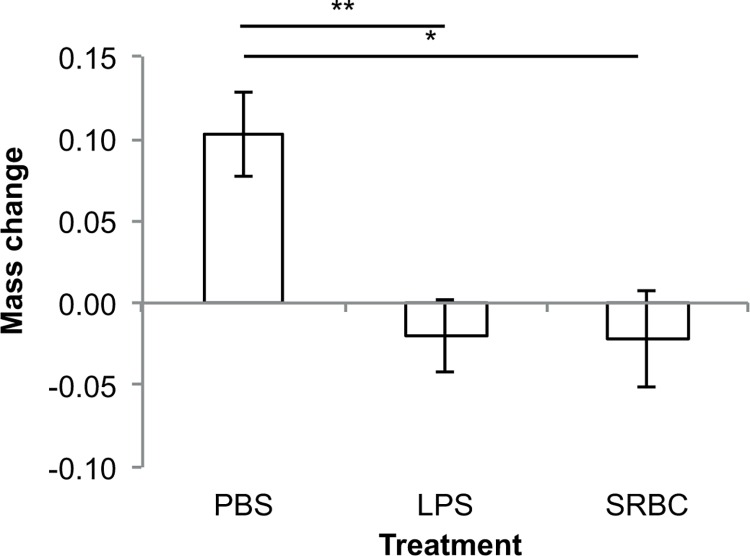
Mass change between the day of the injection (day 0) and 7 days after injection. Values show predicted means (in g) with standard errors (** indicates p≤0.01 and *** indicates p≤0.001).

LPS-injected fish displayed a significantly greater metabolic scope over the course of the experiment than sham-injected controls (GLMM, treatment: F_1,36_ = 1.44, p = 0.24, time: F_2,36_ = 0.8, p = 0.46, treatment × time: F_2,36_ = 3.3, p = 0.05, initial mass: F1_,36_ = 4.7, p = 0.037; Table 1; Fig 1C). Separate analyses of each time point revealed a significant difference between LPS-challenged and control fish 48h after treatment (24h: t_36_ = -1.01, p = 0.32; 48h: t_36_ = 2.32, p = 0.026; 7 days: t_36_ = 1.04, p = 0.31). Although SBRC-injected fish displayed higher metabolic scopes than controls 7 days post-injection, the effect of treatment on metabolic scopes at that time point was not significant (GLM, treatment: F_2,25_ = 0.8, p = 0.443, initial mass: F_1,25_ = 5.7, p = 0.025; Table 1; Fig 2C), with no significant differences detected in any of the pairwise comparisons (all p>0.1). There was a significant effect of treatment on mass change over the course of the experiment (GLM, treatment: F_2,25_ = 7.3, p = 0.003, initial mass: F_1,25_ = 5.0, p = 0.034; Fig 3), with mass change differing significantly between LPS- and control fish (t = -3.7, p = 0.001) and between SRBC- and control fish (t = 2.8, p = 0.010). LPS-fish lost on average over 4% of their body mass over the 7 days of the experiment (initial mass = 0.73±0.21g; final mass = 0.70±0.18g), but SRBC and control-fish increased their body mass by 5% and 8%, respectively (SRBC: initial mass = 0.42±0.14g, final mass = 0.44±0.17g; control: initial mass = 0.90±0.24g, final mass = 0.97±0.21g).

## Discussion

Our results show that experimental challenges with an inert antigen can give rise to changes in resting and maximal metabolic rates, with measurable consequences for metabolic scopes and hence the overall amount of ATP available to aerobically-challenging activities other than those at rest. Resting and maximal metabolic rates and the metabolic scope were significantly increased relative to controls in LPS-challenged fish only. Furthermore, immune-challenged fish gained significantly less body mass over the course of the experiment than controls, with LPS-fish actually losing body mass. Our results indicate that oxidative metabolic capacities can be increased in immune-challenged individuals, such that these individuals actually produce more ATP than would be needed for engaging in aerobically-challenging activities in the absence of an immune challenge.

The higher resting metabolic rate displayed by LPS-challenged individuals relative to controls corroborates the existence of an energetic cost associated with the immune response to LPS. LPS are immunogenic molecules found in the cell wall of gram-negative bacteria that can rapidly trigger a strong inflammatory response without causing infection [47]. In endotherms, injections with *E*. *coli* LPS are commonly used to assess the acute phase response, which occurs within hours of challenge and includes changes in body temperature (e.g., fever) and the expression of sickness behaviours [48–51]. Responses to LPS have been indirectly shown to be costly. For example, LPS-challenged individuals displayed decreased food intake, activity and growth, and exhibited reduced reproductive output [15, 16, 50, 52–54]. Furthermore, direct energetic costs of LPS injections have been demonstrated as a 10 and 20% increase in the resting metabolic rates of zebra finch and rats, respectively [16, 55]. In fish, LPS has been shown to induce a depletion of liver glycogen levels in yearling coho salmon (*O*. *kisutch*) and rainbow trout [56], and it is likely to be a potent agent of anorexia in gold fish (*Carassius auratus auratus*) [57]. Our results verify the metabolic cost of a response to LPS in fish, which can be maintained over the course of a week, but is higher 24h than 7 days post-challenge.

On the other hand, the lack of significant difference in resting metabolic rates between SRBC-treated and control fish suggests either that the cost of immunity to SRBC is negligible or that it becomes detectable later than 7 days post-challenge. SRBC is a T cell-dependent antigen commonly used to assay humoral immune responses, and anti-SRBC antibodies reach a peak 10 and 12 days after injection in brown trout and Mozambique tilapia, respectively [41, 42]. SRBC-injections were previously found to produce a 8.5% increase in the basal metabolic rate of collared doves 7 days after challenge [24], yet they did not affect body mass and molting in house sparrows (*Passer domesticus*) [58]. Similarly, SRBC-injected greenfinches (*Carduelis chloris*) were shown to exhibit reduced activity, but no mass loss, 4 and 8 days post-challenge [59], suggesting that SRBC induces only a mild sickness response. While the metabolic costs and consequences of the immune response mounted by mosquitofish against SRBC still remain to be determined, the fact that SRBC-fish gained significantly less mass over the course of the experiment than controls suggests that a challenge with SRBC induces measurable energetic costs the first week post-injection.

Immune-challenged mosquitofish did not display a decreased metabolic scope relative to saline-injected controls, indicating that immune functioning did not give rise to a trade-off at the level of ATP production and use. In fact, not only were metabolic scopes not decreased in LPS-injected fish, but they were actually greater than those of controls, indicating that individuals actually boosted their levels of energy available to physical activity. This increase in metabolic scope was significant only at 48h post-injection in LPS-fish, indicating that this process is not immediate and may only be transitory. One explanation for such overcompensation is that immune-challenged fish require more ATP to sustain swimming than saline-injected controls. LPS has indeed been shown to decrease the efficiency of carbohydrate catabolism by skeletal muscles, that is the ratio between ATP use and power output, giving rise to greater oxygen consumption during activity, and to stimulate muscle wasting leading to muscle dysfunction [60–62]. In fish, white skeletal muscle is mostly anaerobic and used primarily in burst swimming, while red skeletal muscle is aerobic and involved in sustained swimming speed [63]. Gilthead seabream (*Sparus aurata*) challenged with LPS displayed strong transcriptomic responses in their white and red skeletal muscles 24h and 72h post-injection [11]. Protein synthesis and carbohydrate catabolism were strongly increased in white muscle 24h post-LPS administration, which suggested in part that LPS may initially stimulate energy production through glycolysis; these patterns were, however, reversed at 72h possibly indicating muscle atrophy. Genes involved in aerobic metabolism and protein synthesis, on the other hand, were up-regulated 72h post-challenge in red muscle [11]. Similar increases in carbohydrate metabolism were observed in the fast muscle fibres of rainbow trout following challenge with LPS [64]. Whether the increased aerobic metabolism that we detected in mosquitofish 48h post-immune challenge with LPS, allowed fish to swim at an equivalent speed or faster than control fish was not explored in this study. The capacity to increase aerobic metabolism may be positively selected if it facilitates parallel increases in two or more aerobically-demanding activities (e.g., immunity and locomotion), and when simultaneous investment in each activity maximizes fitness [65, 66]. Future work is required to better understand the consequence of immune-associated increases in metabolic scope on locomotor performance (e.g., swim speed and duration) as well as its evolutionary significance for individual fitness.

While immune functioning did not give rise to a trade-off in terms of metabolic scope, the lowered relative body mass of immune-challenged fish at the end of the experiment suggests that limited acquired food resources, or that impaired food assimilation or mitochondrial use of those resources [67], may instead drive trade-offs between immunity and other aerobic traits. For example, inflammatory responses have indeed been found to cause mitochondrial dysfunction [68] and a lower efficiency of mitochondria as a result of proton leak, for example, would mean that greater amounts of substrate and ultimately food or energy reserves have to be oxidised to achieve a given ATP output [67]. Fish that were challenged with LPS- or SRBC- both exhibited reduced body mass gain relative to controls over the 1 week duration of the experiment, with LPS-fish actually loosing mass and SBC-fish gaining >4 times less mass than controls. Divergence in mass change between SRBC- and LPS-fish is likely to stem, in part, from LPS-induced adaptive anorexia, which may have prevented a compensation of the energetic costs of immunity through greater food intake [57]. Anorexia is, indeed, a host defence mechanism against bacterial infections [69] and a typical component of the sickness response to LPS [16, 52], but not SRBC [58, 59].

Our data show that plasticity in oxidative metabolism is, at least, a short term response to an immune challenge that can increase the fitness of individuals by maintaining locomotor performance, reproductive activity, and similar aerobically-demanding functions. The benefits of such up-regulated ATP production, however, are expected to diminish with muscle loss and as body condition decreases below critical levels.
